# The obesity conundrum in sepsis

**DOI:** 10.1186/s12871-017-0434-z

**Published:** 2017-10-25

**Authors:** Pauline Yeung Ng, Matthias Eikermann

**Affiliations:** 1 Adult Intensive Care Unit, Queen Mary Hospital and The University of Hong Kong, Pok Fu Lam, Hong Kong; 2 Department of Anesthesia, Critical Care and Pain Medicine, Beth Israel Deaconess Medical Center and Harvard Medical School, 330 Brookline Avenue, Boston, MA USA

**Keywords:** Obesity, Overweight, Sepsis, Septic shock, Obesity paradox, Mortality, ICU, Meta-analysis

## Abstract

While the long-term negative effects of obesity on health is a well-studied phenomenon, its effects on acute illnesses seem to be the contrary. Several studies have indicated the possibility of an ‘obesity paradox’ in sepsis – where overweight and obese patients have better outcomes than normal weight patients. These meta-analyses including large numbers of patients across different countries raised an interesting but debatable topic. Results from meta-analyses of observational studies should be interpreted with caution, and a prove of association not be mistaken as prove of causality. Limitations common to such studies include inadequate adjustment for confounding and selection bias. More rigorous investigations to clarify any causal relationship between obesity and mortality in sepsis are needed.

## Background

While the long-term negative effects of obesity on health is a well-studied phenomenon, its effects on acute illnesses seem to be the contrary. Many studies have indicated the possibility of an ‘obesity paradox’ – where overweight and obese patients have better outcomes than normal weight patients, in acute medical conditions such as acute coronary syndrome [1] and acute respiratory distress syndrome [2].

**Table 1:** Factors supporting and refuting the obesity paradox in sepsis

Possible pathophysiological mechanisms of the obesity paradox	Possible biases in studies examining the obesity paradox
Higher metabolic reserves in acute catabolic illnesses	Inadequate adjustment for confounding factors e.g. smoking
Secretion of anti-inflammatory mediators by adipose tissue e.g. leptin, soluble tumor necrosis factor-receptor-2	Selection bias of patients with less severe sepsis in obese populations
Hemodynamic benefits of renin-angiotensin system activation	Protective effect limited to certain subpopulations e.g. older patients with comorbidities
High-density lipoproteins bind bacterial lipopolysaccharide	Relatively restrictive administration of medications in obese patients due to a non-weight based principle
Obesity and resulting obstructive sleep apnea contributes to ischemic preconditioning	Misclassification of patients due to inaccurate BMI measurements
	Decreased BMI may be due to sarcopenia

In a recently-published study in BMC Anesthesiology, Wang et al. analyzed the association between body weight and outcomes in septic patients in a meta-analysis of eight studies [3]. In their study, the authors concluded that overweight, but not obese or morbidly obese patients had lower adjusted mortality rates than normal weight patients. Although their work seems to be another obvious example of the obesity paradox and the benefits of having higher metabolic reserves in acute illnesses, the results must be interpreted with caution.

## Main text

In 1997, the World Health Organization defined weight categories based on body mass index (BMI) cutoff values – underweight (BMI of <18.5), normal weight (BMI of 18.5 to <25), overweight (BMI of 25 to <30), and obesity (BMI of ≥30) [4]. Recent studies have estimated that roughly 30% of the population in the United States are overweight, and another 30% are obese [5]. Despite the widespread public health initiatives to reduce the prevalence of obesity and its related comorbidities, studies in the last decade have begun to show the possible protective effects of overweight in specific diseases – a phenomenon coined as the ‘obesity paradox’ [1, 2, 6, 7]. Our group also reported a similar observation: that in patients with obstructive sleep apnea – who are often obese - perioperative in-hospital mortality tends to be lower [8].

Two recent meta-analyses studied the obesity paradox in patients with sepsis. Pepper et al. included four retrospective and two prospective studies to show that overweight or obese BMIs reduced adjusted mortality in adults admitted to the intensive care unit with sepsis, severe sepsis, or septic shock [9]. In a more recent analysis, Wang and co-authors pooled data from three of the previously included studies with five additional studies, including 9696 patients, and concluded that overweight, but not obesity or morbid obesity, was associated with lower mortality in sepsis patients [3].

Several pathophysiological mechanisms have been postulated to explain the obesity paradox in critically ill patients with sepsis (Table 1). First, obesity may be associated with having higher metabolic reserves, which is beneficial in acute illnesses that are catabolic. Secondly, adipose tissues may modulate the inflammatory response by secreting anti-inflammatory mediators such as leptin [10] and soluble tumor necrosis factor-receptor-2 [11]. Also, heightened renin-angiotensin system activation may confer hemodynamic advantages in sepsis [12].

However, meta-analyses of observational studies should be interpreted with caution, and a prove of association not be mistaken as prove of causality. The inclusion of retrospective studies poses the problem of inadequate adjustment for confounding. In particular, the underrepresentation of smokers in overweight and obese populations may bias lower mortality rates in these patient groups. Overweight and obesity may also have influenced the decision to admit otherwise less critically ill patients with sepsis, or patients with more easily-treatable infections, to intensive care, resulting in a selection bias [13]. Moreover, the protective effects of overweight may be limited to certain subpopulations, for example older and patients with existing comorbidities – in fact, the mean age of one included study was more than 70 years [14]. The administration of intravenous fluids [13] by a non-weight based principle may be comparatively restrictive and benefit patients with higher BMIs [13]. Lastly, inaccuracies in measuring BMI during acute illness, for example the presence of tissue edema from fluid resuscitation, may have created misclassification of normal weight individuals into the overweight category.

## Conclusion

Globally, the incidence of sepsis and the number of deaths related to sepsis are increasing. There is a pressing need to identify factors associated with increased sepsis-related mortality to better prognosticate patient outcomes and allocate intensive care resources. These meta-analyses including large numbers of patients across different countries raised an interesting but debatable example of the obesity paradox in sepsis. More rigorous investigations to clarify any causal relationship between obesity and mortality in sepsis are needed.

## Abbreviation

BMI: Body mass index
